# Antifungal Potential of Essential Oil Chemotypes of *Eugenia uniflora* Against Phytopathogens

**DOI:** 10.1002/cbdv.202502836

**Published:** 2026-02-25

**Authors:** Ygor Nunes Moreira, Eduardo Barros Duarte‐Junior, Igor Sampaio Fontes, Elisabeth Alves Duarte Pereira de Medeiros, Camila da Silva Barbosa Pereira, Diego da Paixão Alves, Durval Reis Mariano‐Junior, Rosana Santos Cavalcante, Pedro Corrêa Damasceno‐Junior, André Marques dos Santos, Marco Andre Alves de Souza

**Affiliations:** ^1^ Graduate Program in Environmental and Forestry Sciences, Institute of Forests Universidade Federal Rural Do Rio de Janeiro (UFRRJ) Seropédica RJ Brazil; ^2^ Graduate Program in Chemistry, Institute of Chemistry Universidade Federal Rural do Rio de Janeiro (UFRRJ) Seropédica RJ Brazil; ^3^ Department of Biochemistry, Institute of Chemistry Universidade Federal Rural do Rio de Janeiro Seropédica RJ Brazil; ^4^ Department of Agrotechnology and Sustainability, Institute of Agronomy Universidade Federal Rural Do Rio de Janeiro Seropédica RJ Brazil

**Keywords:** anthracnose, biological activity, natural product, phytopathogens, surinam cherry

## Abstract

This study investigated the chemical composition and antifungal activity of essential oils (EOs) from five chemotypes of *Eugenia uniflora* (*E. uniflora*) collected in the state of Rio de Janeiro, Brazil. GC–FID and GC–MS analyses revealed major compounds such as curzerene, selina‐1,3,7(11)‐trien‐8‐one, its epoxide, and spathulenol, indicating significant chemical variability. In vitro assays demonstrated that EOs from selected chemotypes exhibited strong inhibitory effects against the phytopathogens *Colletotrichum gloeosporioides* and *Pestalotia* sp., with inhibition rates comparable to the fungicide tebuconazole. IC_50_ values ranged from 0.01 to 0.09 mg mL^−1^. These findings reinforce the potential of *E. uniflora* as a natural source of bioactive compounds and support further exploration of its EOs for sustainable plant disease management. The observed intraspecific chemical diversity may influence biological effects and highlights the importance of chemotype selection in the development of botanical fungicides.

## Graphical abstract

This study investigates the chemical profile and antifungal activity of Eugenia uniflora essential oils. The major constituents identified were selina‐1,3,7(11)‐trien‐8‐one and curzerene. The oils showed significant inhibitory effects against the phytopathogens Colletotrichum gloeosporioides and Pestalotia sp. These results emphasize the potential of E. uniflora metabolites as sustainable natural alternatives for fungal disease management in agriculture.

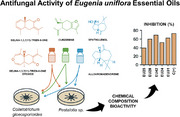

## 1 Introduction

Fruit production—including bananas, papayas, oranges, apples, and watermelons—plays a significant role in the Brazilian economy and global market, contributing to the local development of small‐ and medium‐scale farmers [1, 2]. Despite their high commercial value, these fruits are particularly susceptible to infections caused by phytopathogenic fungi during both pre‐harvest and post‐harvest stages [3].

Post‐harvest losses result from the cumulative degradation of products due to delays in commercialization or consumption. This deterioration occurs across multiple stages, including production, storage, transportation, and marketing. In the case of diseases caused by phytopathogenic fungi, such losses can reach up to 80% of the final market value of agricultural products [3].

One of the most important agricultural diseases is anthracnose, caused by the fungus *Colletotrichum gloeosporioides*, which affects a wide range of tropical fruits. The main symptoms include the appearance of dark spots and an unpleasant odor, typically manifesting after harvest in fruits that were previously infected. Among the fruits most affected by anthracnose are mango, papaya, pitaya, and avocado [4]. Another agriculturally significant disease is Pestalotia spot, caused by fungi of the genus *Pestalotia* sp., whose primary hosts include strawberries and certain forest species such as cedar [5]. The typical symptoms of this infection are dark brown lesions on leaves, petioles, stolons, sepals, and fruits, leading to a significant reduction in productivity [6].

With the aim of promoting sustainable, pesticide‐free, and effective control strategies for tropical fruit crops, the use of essential oils (EOs) has emerged as a promising approach to combat agricultural diseases and pests. In this context, the Surinam cherry (*Eugenia uniflora* L.) has been reported in the literature as a species with potential biological activity against fungi, bacteria, and protozoa of medical interest [7, 8, 9].

Although the antifungal activity of *E. uniflora* essential oil is well‐documented against human pathogens, such as *Candida* spp. (e.g., *C. albicans*, *C. krusei*, and *C. tropicalis*), and certain phytopathogens like *Thielaviopsis basicola* and *Fusarium oxysporum* [10, 11, 12]; however, to our knowledge, there are no published reports of antifungal activity of *E. uniflora* essential oil specifically against *Colletotrichum gloeosporioides* or *Pestalotia* sp. To date, no comparative study has addressed the antifungal potential of distinct *E. uniflora* chemotypes against these specific phytopathogens.

Thus, *E. uniflora* represents a promising candidate for further studies on its fungicidal action against agriculturally important pathogens. Accordingly, the present study aimed to evaluate the inhibitory potential of *E. uniflora* EOs on the mycelial growth of *Colletotrichum gloeosporioides* and *Pestalotia* sp.

## 2 Results and Discussion

The analysis of essential oils from *Eugenia uniflora* plants (EU24, EU25, EU28, EU31, and EU42) revealed a diversity of compounds, including hydrocarbon sesquiterpenes (SH) and oxygenated sesquiterpenes (SO), as shown in Table 1. Among the identified compounds, curzerene—a Cope rearrangement product of furanodiene [13, 14]—was the major constituent in EU24 (27.7%) and EU25 (59.0%). Selina‐1,3,7(11)‐trien‐8‐one was predominant in the EOs of EU28 (42.4%) and EU31 (51.7%), whereas spathulenol was the main component in EU42 (30.3%).

**TABLE 1:** Main compounds found in the essential oils of *Eugenia uniflora*.

Compounds		LRIC	LRIL	EU24	EU25	EU28	EU31	EU42
				——– Relative proportion (%) ——‐				
Myrcene	MH	992	988	2.59	—	—	—	—
Linalool	OM	1107	1095	—	—	—	0.49	—
ρ‐Cymene	MH	1028	1020	2.1	—	—	—	—
(E)‐β‐Ocimene	MH	1050	1044	1.45	—	—	—	—
(E)‐Patchenol	OM	1331	1328	—	—	—	—	3.3
β‐Elemene	SH	1392	1389	7.4	5.9	3.1	2.5	—
(E)‐Caryophyllene	SH	1422	1417	7.9	—	1.9	1.7	8.3
γ‐Elemene	SH	1435	1434	1,5	5.9	2.2	0.6	—
Allo‐Aromadendrene	SH	1464	1458	—	2.4	1.7	—	17.0
9‐epi‐(E)‐Caryophyllene	SH	1465	1464	—	4.14	—	—	—
Germacrene D	SH	1486	1480	4.16	—	0.68	1.03	—
β‐Selinene	SH	1493	1489	3.03	—	—	—	—
Ledene	SH	1495	1496	—	—	1.43	—	—
Viridiflorene	SH	1496	1496	—	—	1.43	—	—
Bicyclogermacrene	SH	1500	1500	—	—	2.1	0.89	—
Curzerene*	OS	1503	1499	27.7	59.0	—	—	—
α‐Muurolene	SH	1518	1500	—	—	—	—	1.33
δ‐Amorphene	SH	1525	1511	—	—	—	—	3.97
Germacrene B	SH	1567	1559	19.7	7.9	4.4	5.3	—
Spathulenol	OS	1590	1577	1.9	4.9	0.9	0.4	30.3
Caryophyllene Oxide	OS	1592	1582	1.6	—	—	—	5.2
Globulol	OS	1597	1590	1.58	3.79	1.26	—	—
Viridiflorol	OS	1607	1592	—	—	0.73	—	—
β‐Copaen‐4‐α‐ol	OS	1638	1590	—	—	—	—	5.0
Selina‐1,3,7(11)‐trien‐8‐one	OS	1643	1632	—	—	42.4	51.7	—
Allo‐Aromadendrene Epoxide	OS	1648	1639	—	—	—	—	2.27
epi‐α‐Muurolol	OS	1660	1640	—	—	—	—	4.03
α‐Cadinol	OS	1672	1652	—	—	—	—	4.41
Selin‐11‐en‐4‐α‐ol	OS	1674	1658	—	—	—	0.54	—
Furanodiene	OS	1710	1699	4.9	5.7	—	—	—
Germacrone	OS	1711	1693	—	—	—	0.5	—
Selina‐1,3,7(11)‐trien‐8‐one Epoxide	OS	1763	1746	—	—	32.9	33.2	1.4
Total Monoterpenes				6.14	0.0	0.0	0.49	3.3
Total Sesquiterpenes				81.37	99.63	97.13	98.36	83.21
Total				87.51	99.63	97.13	98.85	86.51

*Note*: Relative proportions (%), calculated linear retention indices (LRIC), and literature retention indices (LRIL) are presented.

Abbreviations: MH: monoterpene hydrocarbon; OM: oxygenated monoterpene; OS: oxygenated sesquiterpene; SH: sesquiterpene hydrocarbon. ^*^Curzerene is a Cope rearrangement product derived from furanodiene [12].

The compound selina‐1,3,7(11)‐trien‐8‐one epoxide was primarily detected in EU28 (32.9%) and EU31 (33.2%). Additionally, β‐elemene and (*E*)‐caryophyllene were identified in the EOs of several plants, with higher abundances in EU24, while *allo*‐aromadendrene was found in greater proportion in EU42 (17.0%). These results highlight the chemical variability of *E. uniflora* EOs. Such variability may influence the biological activity and potential applications of these oils.

The chemical diversity observed in the EOs of *E. uniflora* (Table 1) suggests the potential for varied biological properties, particularly antifungal activity, as demonstrated in Table 2. The inhibitory effects of EOs from EU31, EU42, EU25, EU24, and EU28 were evaluated against *C. gloeosporioides* and *Pestalotia* sp. at different concentrations (0.05, 0.10, 0.44, 0.88, 1.75, and 3.50 mg mL^−1^) and compared with the positive control, tebuconazole (Table 2).

**TABLE 2:** Inhibitory effects (%) of treatments with *Eugenia uniflora* essential oils on *Colletotrichum gloeosporioides* and Pestalotia sp.

	*Colletotrichum gloeosporioides*			*Pestalotia* sp.		
Treatments	EU31	EU42	EU25	EU31	EU24	EU28
———‐ mg mL^−1^ ———‐	——————————‐ Inhibition (%) ——————————‐					
0.00	0.0 d	0.0 f	0.0 d	0.0 d	0.0 c	0.0 d
0.05	16.4 d	40.8 e	13.6 d	42.6 c	67.7 b	60.5 c
0.10	53.0 c	40.7 e	42.6 c	67.3 b	65.9 b	82.8 b
0.44	66.0 bc	57.3 cd	65.8 b	88.7 a	85.5 a	87.0 ab
0.88	65.3 bc	71.1 b	70.4 ab	84.7 ab	87.1 a	86.4 ab
1.75	73.0 ab	57.0 d	76.2 ab	85.2 ab	87.2 a	87.9 ab
3.50	73.2 ab	71.0 bc	77.7 ab	88.2 a	86.1 a	89.2 a
Control (+)	87.2 a	87.2 a	87.2 a	87.3 a	87.3 a	87.3 ab
*F*	83.75	73.09	68.77	494.68	306.28	705.50
(DFn. DFd)	(7, 32)	(7, 32)	(7, 32)	(7, 32)	(7, 32)	(7, 32)
*p*	<0.0001	<0.0001	<0.0001	<0.0001	<0.0001	<0.0001

*Note*: Inhibition percentages were adjusted based on the control without essential oil. EU31, EU42, EU25, EU24, and EU28 correspond to essential oils from different chemotypes of *E. uniflora*. Different letters indicate statistically significant differences among treatments according to Tukey's test (*p* ≤ 0.05).

Abbreviations: PDA: potato dextrose agar; DMSO: dimethyl sulfoxide; Control (+): Folicur (tebuconazole) at 0.25 mg mL^−1^.

The EOs showed significant inhibition at concentrations above 0.05 mg mL^−1^ against *C. gloeosporioides*. Concentrations of 0.44 mg mL^−1^ for EU31, EU42, and EU25 resulted in inhibitions of 66.0%, 57.3%, and 65.8%, respectively. It is important to highlight that EU31 and EU25 presented results similar to the positive control with tebuconazole at concentrations of 1.75 and 0.88 mg mL^−1^, respectively (Table 2).

For *Pestalotia* sp., treatments with 0.44 mg mL^−1^ of the EOs from EU31, EU24, and EU28 resulted in inhibitions of 88.7%, 85.5%, and 87.0%, respectively, similar to the positive control, which showed an inhibition of 87.3% (Table 2).

Additionally, the inhibition values (%) were subjected to dose–response analysis to present the results shown in Table 3, which displays the median inhibitory concentration (IC_50_) of *E. uniflora* essential oils against the fungi *C. gloeosporioides* and *Pestalotia* sp. For *C. gloeosporioides*, the EOs from EU31, EU42, and EU25 showed IC_50_ values of 0.07, 0.05, and 0.09 mg mL^−1^, respectively. Against *Pestalotia* sp., the EOs from EU31, EU24, and EU25 demonstrated greater efficacy, with IC_50_ values of 0.02, 0.01, and 0.04 mg mL^−1^, respectively.

**TABLE 3:** Median inhibitory concentration (IC_50_) of *Eugenia uniflora* essential oils against *Colletotrichum gloeosporioides* and *Pestalotia* sp.

Essential oils	IC_50_ (±CI) mg mL^−1^	Slope	*R* ^2^	Sy.x
	—————– *Colletotrichum gloeosporioides* —————–			
EU31	0.07 ±0.01	3.37	0.96	5.57
EU42	0.05 ±0.03	0.55	0.92	6.77
EU25	0.09 ±0.02	2.00	0.94	7.35

	—————————‐ *Pestalotia* sp —————————			
EU31	0.02 ±0.01	1.50	0.99	1.89
EU24	0.01 ±0.01	0.61	0.97	5.05
EU28	0.04 ±0.01	3.27	0.99	1.28

*Note*: IC_50_ values (mean ± confidence interval), slope of the dose–response curve, coefficient of determination (*R* ^2^), and standard deviation of residuals (Sy.x) are presented. IC_50_: concentration required to inhibit 50% of fungal growth.

The EOs of *E. uniflora* exhibited notable chemical variability, with major constituents including curzerene, selina‐1,3,7(11)‐trien‐8‐one, and spathulenol (Table 1). The presence of distinct chemotypes within the species, indicative of intraspecific chemical variability, suggests the potential for varied biological effects. This was confirmed in the present study by the observed inhibitory activity against the fungi *C. gloeosporioides* and *Pestalotia* sp (Tables 2 and 3). Previous studies have also reported the occurrence of different chemotypes in *E. uniflora*, highlighting curzerene [15], selina‐1,3,7(11)‐trien‐8‐one [16], and germacrene B [17] as prominent constituents.

The findings from the inhibition assays confirm the strong antifungal activity of the EOs, particularly those tested against *Pestalotia* sp., which exhibited inhibition levels comparable to the positive control, tebuconazole (Table 2).

Chemotypes EU24, EU31, and EU25 demonstrated significant antifungal activity against *Pestalotia* sp., exhibiting IC_50_ values ranging from 0.01–0.02 mg mL^−1^, which suggests their potential application as natural fungicides. This activity is comparable to promising results reported in the literature for de *Eugenia* species. We believe that the efficacy of these chemotypes can be correlated with their major chemical constituents. EU31 is characterized by selina‐1,3,7(11)‐trien‐8‐one, EU25 by furanodiene, and EU24/EU25 by their respective oxygenated sesquiterpenes. The lipophilic nature of these compounds’ facilities their penetration through the fungal cell wall, interacting with the ergosterol‐containing plasma membrane. This interaction often results in the disruption of membrane integrity and leakage of vital intracellular constituents [18]. Furthermore, the complex mixture of the essential oil likely provides a synergistic “entourage effect”, where minor constituents potentiate the activity of the main markers.

Although studies on the activity of *E. uniflora* EOs against phytopathogenic fungi are limited, some have reported moderate effects of oils rich in selina‐1,3,7(11)‐trien‐8‐one and its epoxide against fungi of the genus *Candida*. Previous reports indicate inhibition of *Candida albicans* at concentrations of 4.09 mg mL^−1^ [10] and 0.21 mg mL^−1^ [19]. Additionally, minimum inhibitory concentrations (MICs) of 0.06 and 0.25 mg mL^−1^ have been reported against *Paracoccidioides brasiliensis* for *E. uniflora* EOs rich in curzerene and selina‐1,3,7(11)‐trien‐8‐one, respectively [17].

The literature therefore highlights the potential of *E. uniflora* essential oil for microbial control, and the present study corroborates these findings by demonstrating its toxicity against phytopathogenic fungi.

The essential oil from *Eugenia uniflora* leaves exhibited strong antifungal activity against *Colletotrichum gloeosporioides* and *Pestalotia* sp. Despite these promising results, further studies are needed to better elucidate the antifungal potential of this native species from Brazilian restinga ecosystems, particularly with a view toward developing botanical pesticides under different conditions and production scales. Such efforts could contribute to the advancement of pesticide‐free agriculture, especially in the context of fruit crop protection.

## 3 Conclusions

The EOs of *Eugenia uniflora* exhibited significant chemical variability and strong antifungal activity against *Colletotrichum gloeosporioides* and *Pestalotia* sp. The results highlight the potential of selected chemotypes as sources of bioactive compounds for the development of botanical fungicides. However, it is important to acknowledge the limitations of this study. While the chemical profiles suggest a membrane‐disruption mechanism, specific molecular targets were not experimentally validated through microscopic or enzymatic assays. Additionally, the potential phytotoxicity of these essential oils on host crops remains to be evaluated to ensure their safety in agricultural applications. Furthermore, extrapolating in vitro efficacy to field scenarios presents challenges, primarily due to the high volatility and environmental instability of EOs. Consequently, future research should prioritize the development of stable formulations (e.g., nanoencapsulation) and the execution of in vivo and field trials to validate the practical applicability of these chemotypes as biofungicides.

## 4 Experimental Section

### 4.1 Isolation and Identification of Phytopathogenic Fungi

The fungus *Colletotrichum gloeosporioides* was isolated from papaya (*Carica papaya* L.). Identification was based on morphological comparison of fungal structures, including mycelia, conidia, conidiophores, and appressoria [20, 21]. The fungus *Pestalotia* sp. was provided by the Mycology and Mycotoxicology Laboratory at Universidade Federal Rural do Rio de Janeiro. Both fungi were maintained on potato dextrose agar (PDA) and incubated in a growth chamber at a controlled temperature of 27 °C.

### 4.2 Plant Collection, Drying, and Identification

Branches and leaves of Surinam cherry (*E. uniflora*) were collected during two expeditions in the North Fluminense region, Rio de Janeiro state, Brazil. The first collection occurred on September 23, 2022, in the municipalities of Rio das Ostras, Macaé, and Campos dos Goytacazes (*n* = 10 specimens). The second collection was performed on November 18, 2022, in Campos dos Goytacazes, São João da Barra, and São Francisco de Itabapoana (*n* = 1 specimen), totalling 20 accessions. Approximately 1 kg of plant material was collected per individual. The plant material was dried at 37 °C for 48 h in a forced‐air circulation oven. A sample from each plant was reserved for identification and deposited in the Herbarium of the Universidade Federal Rural do Rio de Janeiro (Herbarium RBR). The accessions were labelled with the prefix “EU”. Geographic coordinates (GPS) and environmental description was performed by the specialist team at the Herbarium RBR (Department of Botany, Universidade Federal Rural do Rio de Janeiro). Voucher specimen data and deposit numbers can be verified at the herbarium database (https://rbr.jbrj.gov.br/v2/consulta.php). The access codes and corresponding voucher numbers were EU24 (RBR 57579), EU25 (RBR 57580), EU28 (RBR 57583), EU31 (RBR 57586), and EU42 (RBR 57972). From the collected set, five accessions (EU24, EU25, EU28, EU31, and EU42) were selected for detailed chemical and biological characterization. This selection was based on preliminary chromatographic profiles (GC–FID) to ensure the representation of different chemical phenotypes (chemotypes) present in the study area.

### 4.3 Hydrodistillation and Analysis of Essential Oils

EOs were obtained by hydrodistillation for 3 h using a Clevenger‐type apparatus, following a previously described method [22]. The oils were labelled with the same codes as the respective plant samples. Chemical analyses were performed using GC–FID (HP 5890 Series II) and GC–MS (Shimadzu QP‐2010 Plus). Procedures for sample preparation, chromatographic conditions, equipment settings, and compound identification followed previously established protocols and recommendations [22, 23]. Constituent identification was performed using a multi‐parameter approach. Mass spectra were compared against the NIS 2008 library (considering a similarity threshold of ≥ 80%) and visually verified against diagnostic fragmentation patterns described by Adams [24]. Retention indices (RI) were calculated using a homologous series of *n*‐alkanes (C_8_–C_30_). Identification relied on an RI tolerance of ± 10 units for monoterpenes and ± 20 units for sesquiterpenes. Methyl octanoate was used as an internal standard for the normalization of chromatographic peaks. No isolated analytical standards were used for identification in this study.

### 4.4 Assays

### 4.5 Preparation of Treatments

Six treatments with different concentrations of essential oils were prepared using the dilution method in PDA medium, with dimethyl sulfoxide (DMSO) serving as the diluent and carrier. The tested concentrations were 0.05, 0.10, 0.44, 0.88, 1.75, and 3.50 mg mL^−1^. Additionally, a positive control containing tebuconazole (0.25 mg mL^−1^), a negative control with DMSO (0.25 mg mL^−1^), and a control with only PDA medium were included.

### 4.6 In Vitro Antifungal Assay

Sample preparation and assays were conducted in a laminar flow chamber sterilized with 70% ethanol and ultraviolet light. EOs from samples EU31, EU42, and EU25 were evaluated against *Colletotrichum gloeosporioides*, while oils from EU31, EU24, and EU28 were tested against *Pestalotia* sp. Each treatment included five replicates. Petri dishes were incubated for 48 h at 27 °C. The experimental procedure followed the method previously described [25].

### 4.7 Analysis of Results

After the incubation period, Petri dishes were scanned. Images were processed using ImageJ software (V.1.54 g), converted to 8‐bit black‐and‐white format, and subjected to pixel segmentation to differentiate between mycelium areas (white pixels) and the remaining plate surface (black pixels). An appropriate threshold was established to delineate the boundary between colonized and noncolonized areas. Growth inhibition was calculated based on the total growth area. No data restrictions were applied. Data normality and homoscedasticity were verified using Shapiro–Wilk and Bartlett's test (*p* > 0.05), respectively. Results were analyzed by one‐way ANOVA followed by Post‐hoc Tukey's test using GraphPad Prism software (Version 8). The median inhibitory concentration (IC_50_) was calculated using nonlinear regression analysis. The data were fitted to a four‐parameter logistic model (log(inhibitor) versus normalized response – Variable slope) described by the equation: *Y = Bottom + (Top – Bottom) / (1 + 10^(LogIC50 – X) x HillSlope^)*, where *Y* represents the percentage of inhibition and *X* represents the logarithm of the essential oil concentration. The assay was performed in quintuplicate (*n* = 5), and the goodness of fit was assessed by the coefficient of determination (*R^2^*). The 95% confidence intervals (95% CI) were reported to demonstrate the precision of the estimated IC_50_ values. The median inhibitory concentration (IC_50_), and graphical representations were performed using GraphPad Prism 8 software. Overall, the analysis procedures followed the methodology previously proposed [25].

## Author Contributions

**Ygor Nunes Moreira**: writing – original draft, validation, resources, methodology, investigation, formal analysis. **Eduardo Barros Duarte‐Junior**: writing – original draft, methodology, investigation. **Igor Sampaio Fontes**: writing – original draft, methodology, investigation. **Elisabeth Alves Duarte Pereira de Medeiros**: writing – original draft, methodology, investigation, supervision. **Camila da Silva Barbosa Pereira**: writing – original draft, methodology, investigation, supervision. **Diego da Paixão Alves**: methodology, investigation, supervision, formal analysis. **Durval Reis Mariano‐Junior**: investigation, supervision, formal analysis. **Rosana Santos Cavalcante**: investigation, supervision, formal analysis. **Pedro Corrêa Damasceno‐Junior**: writing – original draft, methodology, investigation. **André Marques dos Santos**: writing – review and editing, supervision, investigation, funding acquisition, formal analysis. **Marco Andre Alves de Souza**: writing – review and editing, supervision, resources, project administration, investigation, funding acquisition, formal analysis, conceptualization, data curation.

## Conflicts of Interest

The authors declare no conflicts of interest.

## Data Availability

The data that support the findings of this study are available from the corresponding author upon reasonable request.
